# Optimizing Reservoir Computers for Signal Classification

**DOI:** 10.3389/fphys.2021.685121

**Published:** 2021-06-18

**Authors:** Thomas L. Carroll

**Affiliations:** US Naval Research Lab, Washington, DC, United States

**Keywords:** reservoir computer, machine learning, neuromorphic, nonlinear dynamics, neuron

## Abstract

Reservoir computers are a type of recurrent neural network for which the network connections are not changed. To train the reservoir computer, a set of output signals from the network are fit to a training signal by a linear fit. As a result, training of a reservoir computer is fast, and reservoir computers may be built from analog hardware, resulting in high speed and low power consumption. To get the best performance from a reservoir computer, the hyperparameters of the reservoir computer must be optimized. In signal classification problems, parameter optimization may be computationally difficult; it is necessary to compare many realizations of the test signals to get good statistics on the classification probability. In this work, it is shown in both a spiking reservoir computer and a reservoir computer using continuous variables that the optimum classification performance occurs for the hyperparameters that maximize the entropy of the reservoir computer. Optimizing for entropy only requires a single realization of each signal to be classified, making the process much faster to compute.

## 1. Introduction

A reservoir computer is a type of recurrent neural network that is particularly easy to train. A typical reservoir computer is created by connecting a set of nonlinear nodes in a network that includes feedback connections. The first reservoir computers used nodes that were modeled on a hyperbolic tangent function (Jaeger, 2001) or excitable neurons that responded to an input by spiking (Natschlaeger et al., 2002). Unlike most neural networks, the network connections in a reservoir computer never change. Instead, training to a reservoir computer takes place by fitting the signals from the individual nodes to a training signal, usually by a linear fit. Because the network never changes, reservoir computers can be constructed from analog systems in which it is not possible to alter the connections between nodes.

Because the connections between nodes do not change, only the output parameters, training a reservoir computer can be faster than training a conventional neural network. Training a reservoir computer that has *M* nodes requires finding *M* linear fit coefficients. Train by adjusting network connections requires adjusting at least *M*^2^ parameters, and most implementations of neural networks have far more parameters than this. In addition, stability is a concern in recurrent neural network training, so the number useful node activation functions is limited.

Examples of reservoir computers so far include photonic systems (Appeltant et al., 2011; Larger et al., 2012; der Sande et al., 2017), analog circuits (Schurmann et al., 2004), mechanical systems (Dion et al., 2018) and field programmable gate arrays (Canaday et al., 2018). Many other examples are included in the review paper (Tanaka et al., 2019), which describes hardware implementations of reservoir computers that are very fast, and yet consume little power, while being small and light. Reservoir computers have been shown to be useful for solving a number of problems, including reconstruction and prediction of chaotic attractors (Jaeger and Haas, 2004; Lu et al., 2017, 2018; Antonik et al., 2018; Zimmermann and Parlitz, 2018), recognizing speech, handwriting or other images (Jalalvand et al., 2018) or controlling robotic systems (Lukoševičius et al., 2012). Reservoir computers have also been used to better understand the function of neurons in the brain (Stoop et al., 2013). Several groups have been using theory to better understand reservoir computers; in Hart et al. (2020), the authors show that there is a positive probability that a reservoir computer can be an embedding of the driving system, and therefore can predict the future of the driving system within an arbitrary tolerance. Lymburn et al. (2019) study the relation between generalized synchronization and reconstruction accuracy, while Herteux and Räth examine how the symmetry of the activation function affects reservoir computer performance (Herteux and Rath, 2020).

As with any neural network, there are hyperparameters of the nodes that must be optimized to get the best performance from the reservoir computer. Usually some form of nonlinear optimization routine is used. These routines require evaluation of the reservoir computer error for many different hyperparameter combinations. This computation can be slow for reservoir computers used for signal fitting or prediction, but it is even slower for reservoir computers used for classification. To evaluate the performance of a reservoir computer for classification, many realizations of a set of test signals must be generated in order to get good statistics on the probability of making an error in signal classification. In this work, for example, there is a set of four signals to be classified, and 100 test examples for each signal are used, so that for each combination of hyperparameters, the reservoir computer output must be computed 400 times. This large number of computations makes optimization impractical.

In this work, a set of hyperparameters is scanned one parameter at a time, and for each scan, the optimum parameter value is found. Some parameter combinations that might generate better performance may be missed, but it is seen that the best results for classification come when the entropy of the reservoir computer is maximized. Minimizing the error in fitting an input signal would seem to be another target for optimization, but it is found that the best classification performance sometimes comes when the fitting error is large.

Two different types of reservoir computer node are used in this work. The first type of node is a two dimensional ordinary differential equation that resembles a spiking neuron. This model was not created with any particular biological system in mind, but biological systems do consist of spiking neurons, so there may be some biological relevance. It is possible that the results seen for one type of node are only true for that type of node, so the second reservoir computer uses nodes that follow a third order ordinary differential equation.

Signals from the Sprott chaotic systems (Sprott, 1994) were used to test the ability of both node types to classify signals. It was found in a previous work (Carroll, 2021a) that the first four Sprott systems (A,B,C and D) were the hardest to distinguish, so these four systems are used here.

After mentioning the four Sprott systems, the statistic used to calculate the entropy of the reservoir computer is introduced. Next the spiking nodes are defined and their classification performance is measured as several parameters are changed. Following the sections on spiking nodes, the polynomial ordinary differential equation nodes are described and their classification performance is evaluated.

## 2. Reservoir Computers

A reservoir computer may be described by

(1) $$\begin{array}{l} \chi_{i}\left(n+1\right)=f\left(\chi_{i}\left(n\right)\right)+\overset{M}{\underset{j=1}{\sum }}A_{ij}\chi_{j}\left(n\right)+w_{i}s\left(t\right) \end{array}$$

where the reservoir computer variables are the χ_*i*_(*n*), *i* = 1…*M* with *M* the number of nodes, *A* is an adjacency matrix that described how the different nodes in the network are connected to each other, **W** = [*w*_1_, *w*_2_, …*w*_*M*_] describes how the input signal *s*(*t*) is coupled into the different nodes, and *f* is a nonlinear function.

When the reservoir computer was driven with *s*(*t*), the first 1,000 time steps were discarded as a transient. The next *N* time steps from each node were combined in a *N* × (*M* + 1) matrix

(2) $$\begin{array}{l} \Omega =\left[\begin{array}{cccc} \chi_{1}\left(1\right) & \ldots & \chi_{M}\left(1\right) & 1\\ \chi_{1}\left(2\right) & & \chi_{M}\left(2\right) & 1\\ \vdots & & \vdots & \vdots \\ \chi_{1}\left(N\right) & \ldots & \chi_{M}\left(N\right) & 1 \end{array}\right] \end{array}$$

The last column of Ω was set to 1 to account for any constant offset in the fit. The training signal is fit by

(3) $$\begin{array}{l} h\left(t\right)=\Omega \text{C} \end{array}$$

where *h*(*t*) = [*h*(1), *h*(2)…*h*(*N*)] is the fit to the training signal *g*(*t*) = [*g*(1), *g*(2)…*g*(*N*)] and **C** = [*c*_1_, *c*_2_…*c*_*N*_] is the coefficient vector.

The fit coefficient vector is then found by

(4) $$\begin{array}{l} \text{C}=\Omega_{inv}g\left(t\right) \end{array}$$

where Ω_*inv*_ is the Moore-Penrose pseudo-inverse of Ω (Penrose, 1955) and *S*′ is an (*M* + 1) × (*M* + 1) diagonal matrix constructed from *S*, where the diagonal element ${S^{\prime }}_{\;i,i}=S_{i,i}/(S_{i,i}^{2}+k^{2})$, where *k* = 1 × 10^−5^ is a small number used for ridge regression (Tikhonov, 1943) to prevent overfitting.

The training error may be computed from

(5) $$\begin{array}{l} \Delta_{RC}=\frac{\text{std}\left[\Omega \text{C}-g\left(t\right)\right]}{\text{std}\left[g\left(t\right)\right]} \end{array}$$

where std[ ] indicates a standard deviation.

## 3. Sprott Systems

The input signals for this work were the *x* signals from one of the Sprott systems A, B, C, or D (Sprott, 1994). These systems may be described by

(6) $$\begin{array}{l} \text{ A }\left[\begin{array}{l} \dot{x}=y\\ \dot{y}=-x+yz\\ \dot{z}=1-y^{2} \end{array}\right]\text{ B }\left[\begin{array}{l} \dot{x}=yz\\ \dot{y}=x-y\\ \dot{z}=1-xy \end{array}\right]\\ \text{ C }\left[\begin{array}{l} \dot{x}=yz\\ \dot{y}=x-y\\ \dot{z}=1-x^{2} \end{array}\right]\text{ D }\left[\begin{array}{l} \dot{x}=-y\\ \dot{y}=x+z\\ \dot{z}=xz-3y^{2} \end{array}\right]. \end{array}$$

In a previous study using a reservoir computer based on continuous variables, these four Sprott systems were more difficult to distinguish than the other Sprott systems. The Sprott systems were numerically integrated by a 4th order Runge-Kutta integration routine that had a variable step size. The time step for the output of the integration routine was 0.01.

Table 1 lists the Lyapunov exponents for the four Sprott systems used in this work.

**Table 1:** Lyapunov exponents of the Sprott systems A, B, C, and D, from Sprott (1994).

**System**			
A	0.014	0	–0.014
B	0.210	0	–1.210
C	0.163	0	–1.163
D	0.103	0	–1.320

## 4. Entropy Statistic

Besides calculating such statistics as Lyapunov exponents for the reservoir computers, it was useful to characterize the entropy of the reservoir computer. Measuring entropy requires a partitioning of the dynamical system. Xiong et al. (2017) lists a number of ways to do this partitioning, although different partitions can give different results for the entropy. Some of these methods begin with the phase space representation of the dynamical system and then coarse grain the phase space representation to create partitions. Because of the different time scales in the spiking system, coarse graining of the spiking reservoir signals leads to a loss of information; the effect of coarse graining on entropy calculations was examined in Xu et al. (2011). It was found that the permutation entropy method (Bandt and Pompe, 2002) avoided this coarse graining problem because it creates partitions based on the time ordering of the signals. Each individual node time series *r*_*i*_(*t*) was divided into windows of 4 points, and the points within the window were sorted to establish their order; for example, if the points within a window were 0.1, 0.3, –0.1 0.2, the ordering would be 2,4,1,3. Each possible ordering of points in a signal *r*_*i*_(*t*) represented a symbol ψ_*i*_(*t*).

At each time step *t*, the individual node signals were combined into a reservoir computer symbol Λ(*t*) = [ψ_1_(*t*), ψ_2_(*t*), …ψ_*M*_(*t*)]. With *M* = 100 nodes there were potentially a huge number of possible symbols, but the nodes were all driven by a common drive signal, so only a tiny fraction of the symbol space was actually occupied, on the order of tens of symbols for the entire reservoir computer.

If *K* total symbols were observed for the reservoir computer for the entire time series, then the reservoir computer entropy was

(7) $$\begin{array}{l} H=-\overset{K}{\underset{k=1}{\sum }}p\left(\Lambda_{k}\right)\log\left(p\left(\Lambda_{k}\right)\right) \end{array}$$

where *p*(Λ_*k*_) is the probability of the *k*'th symbol.

## 5. Spiking Nodes

The spiking node is a simple 2 dimensional ordinary differential equation

(8) $$\begin{array}{l} \frac{du_{i}}{dt}=T_{C}\left(-u_{i}^{3}+u_{i}\times g\left(v_{i},\phi_{i}\right)\right)\\ \frac{dv_{i}}{dt}=T_{C}\left(\frac{1}{R_{\tau }}\right)\left(W_{i}S_{V}-\gamma v_{i}+\sum_{j=1}^{M}A_{ij}u_{j}\right)\\ g\left(v,\phi \right)=\left\{\begin{array}{l} 0\;v<\phi \\ 1\;v>\phi \end{array}\right\}\\ \text{if }u_{i}>0.5\text{ then }v_{i}=0 \end{array}$$

These nodes were chosen because their simplicity made it easy to understand how their behavior depended on their parameters. The variable *u*_*i*_(*t*) is the fast variable, while *v*_*i*_(*t*) is the slow variable, and the ratio of fast to slow times is set by *R*_τ_. The firing threshold ϕ_*i*_ was different for each node and was set to a value drawn from a uniform random distribution between 1 and 1.1. The random firing thresholds created diversity in the set of nodes. There were *M* = 100 nodes in the reservoir computer.

The input signal *S*_*V*_ is a time series of spikes. The elements of the input vector **W** were chosen from a uniform random distribution between 0 and 1. The adjacency matrix **A** was created by randomly selecting half of its entries and setting them to values drawn from a uniform random distribution between –1 and 1. The diagonal elements of **A** were then set to zero. Because the mean value of **A** was 0, the connections between nodes were evenly divided between excitatory and inhibitory. When the connections were balanced in this way, the inhibitory connections canceled out the excitatory connections and no spiking occurred. In order to get spiking, an offset of 0.5 was added to **A**. After the offset was added, **A** was renormalized to set the spectral radius σ, the largest magnitude of its complex eigenvalues, to 0.5.

The reservoir equations were numerically integrated with a fourth order adaptive step size Runge-Kutta integration routine with a time step of 1.

### 5.1. Input Signals for Spiking Nodes

The input signal *s*(*t*) came from the *x* component of one of the Sprott chaotic systems, either system A, B, C or D. In a previous project using a continuous reservoir computer to classify signals, these were the most difficult of the Sprott systems to distinguish. The Sprott systems were integrated with a time step of 0.01.

The Sprott *x* signals were converted to a time series of spikes. First, the input signal *s*(*t*) was normalized to the signal $\tilde{s}\left(t\right)$ which had a maximum of 1 and a minimum of 0. The signal contains *N* points. A minimum and a maximum period between spikes, *T*_*min*_ and *T*_*max*_, were chosen. For an input signal *s*, a spike time series *S*_*V*_ is produced according to

 *S*_*V*_(i) ← 0 *i* = 1…*N*

 *i*_0_ ← 1

 *i*_1_ ← 1 

 **while** **do**(*i*_0_ < *N*)

    SpikePeriod ←⌊*s*(*i*_0_)(*T*_max_ − *T*_min_)⌋ + *T*_min_

    *i*_1_ ← *i*_0_+SpikePeriod

    *S*_*V*_(*i*_1_) ← 1

    *i*_0_ ← *i*_1_

 **end** **while**

The floor operator ⌊⌋ returns the largest integer that is less than the argument. For all the signals in this work, *T*_*max*_ = 100 and *T*_*min*_ = 10.

**Figure 1:**
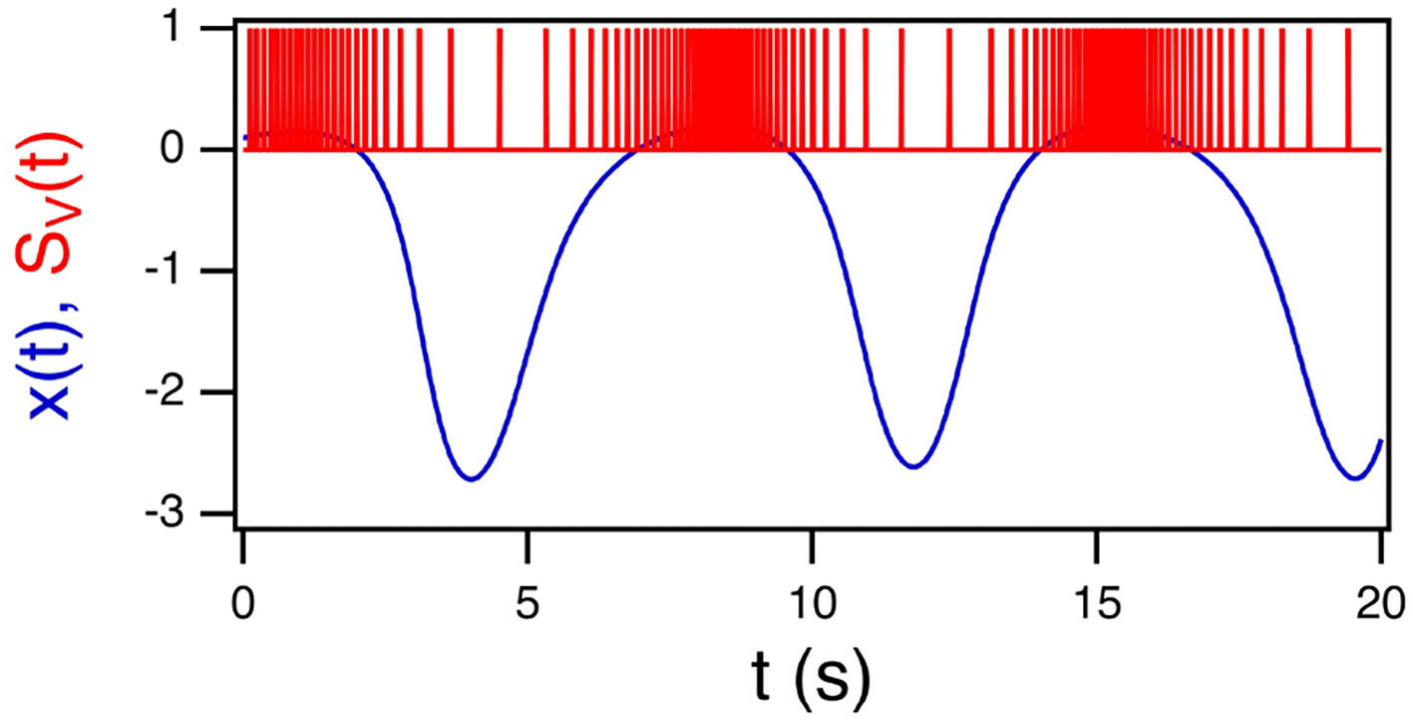
The *x* signal from the Sprott A system in blue and its conversion to a spike time series *S*_*V*_(*t*) in red.

Figure 1 shows the *x* signal from the Sprott A system and its conversion to a time series of spikes.

### 5.2. Training the Spiking System

In the training phase, the reservoir computer was driven with a 100,000 point spiking input signal *S*_*V*_(*t*) from each of the four Sprott systems. The training signal for each instance was the same as the input signal, so the reservoir computer was trained to reproduce *S*_*V*_(*t*). The actual fit signal to *S*_*V*_(*t*) is $g(t)={\sum_{i=1}^{M}c_{i}u_{i}(t)}^{\text{​}}$, where the fit is usually done by a ridge regression to avoid overfitting and the *c*_*i*_'s are the fit coefficients. The training error Δ_*CC*_ was calculated as the cross correlation between *S*_*V*_(*t*) and *g*(*t*):

(9) $$\begin{array}{l} \Delta_{CC}=1-\frac{\overset{N}{\underset{j=1}{\sum }}\left[\left(g\left(j\right)-ḡ\right)\left(S_{V}\left(j\right)-{\bar{S}}_{V}\left(j\right)\right)\right]}{\overset{N}{\underset{j=1}{\sum }}\left(g\left(j\right)-ḡ\right)\overset{N}{\underset{j=1}{\sum }}\left(S_{V}\left(j\right)-{\bar{S}}_{V}\left(j\right)\right)} \end{array}$$

where the overbar operator indicates a mean.

In the training phase, for each Sprott system a set of coefficients **C**_α_, α = A,B,C, or D was produced. For testing the classification procedure, 100 instances of the *x* signal of length 5,000 points were converted to spiking signals and used to drive the reservoir computer of Equation (8). The sets of fit coefficients for the different Sprott systems in the testing phase were designated **K**_β_, β = A,B,C, or D. The fit coefficients may be represented as a matrix

(10) $$\begin{array}{l} \text{K}=\left[\begin{array}{ccc} K_{1,A} & \ldots & K_{M,A}\\ K_{1,B} & \ldots & K_{M,B}\\ K_{1,C} & \ldots & K_{M,C}\\ K_{1,D} & \ldots & K_{M,D} \end{array}\right]. \end{array}$$

The classification error was found by taking the Euclidean difference between the coefficients from the training and testing stages. For example, if β = A, the errors δ_*b*_ were found as

(11) $$\begin{array}{l} \delta_{b}=\sqrt{\overset{M}{\underset{l=1}{\sum }}{\left(K_{l,A}-C_{l,b}\right)}^{2}}b=\text{A,B,C,D} \end{array}$$

where *l* indicates the particular component of the coefficient. If the value of *b* corresponding to the minimum δ_*b*_ is not equal to A, then an error is recorded.

## 6. Spiking Reservoir Classification Performance

The following sections evaluate the fraction of errors in identifying the four Sprott systems as one of the reservoir computer parameters γ, *R*_τ_, or *T*_*C*_ is varied. One parameter is varied while the others are held constant. In many studies of reservoir computers for signal prediction, all the hyperparameters are optimized simultaneously through a nonlinear optimization procedure, but to build up sufficient classification statistics requires many repeat simulations of the reservoir computer for each set of parameters, making optimization computationally very slow. Instead, the classification performance is evaluated for one parameter at a time.

### 6.1. Varying γ

**Figure 2:**
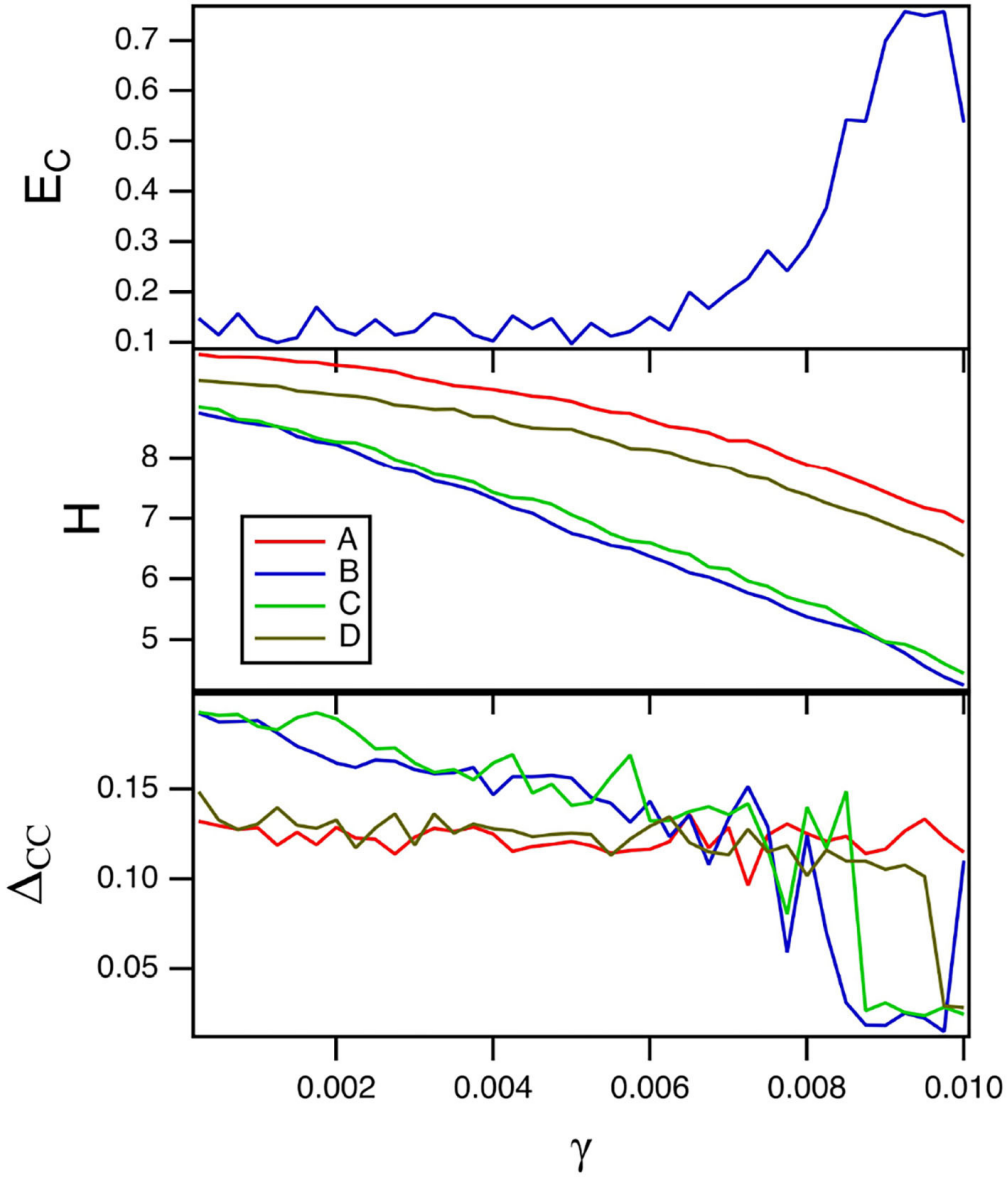
The top plot shows the fraction of errors *E*_*C*_ in identifying which of the four Sprott systems was present, as a function of the damping constant γ for the spiking reservoir computer. The middle plot shows the entropies for the four Sprott systems over the same parameter range. The bottom plot shows the training error Δ_*CC*_. For this plot, *T*_*C*_ = 15 and *R*_τ_ = 36.

The parameter γ is a linear damping parameter in the slow time equation for the spiking nodes (Equation 8). Smaller values of γ mean that the reservoir computer remembers inputs for a longer time. The top plot in Figure 2 shows the fraction of errors *E*_*C*_ in identification as the damping term γ in Equation (8) is varied. For these simulations, *T*_*C*_ = 15 and *R*_τ_ = 36. Because there are four systems, the highest probability of error is 0.75. The minimum error fraction in this plot is about 0.1. The middle plot shows the entropy for the reservoir computer driven by the four different Sprott systems over the same parameter range. Figure 2 shows that larger entropies correspond to lower classification errors, which is expected from Crutchfield and Young (1990) and Langton (1990).

The training error Δ_*CC*_ when each of the four Sprott systems drive the spiking reservoir computer is plotted in the bottom plot in Figure 2. The training error is large when the error in identifying the four Sprott systems is small. This is even more surprising because the entropy *H* for the spiking reservoir computer is larger when the training error is large. Theories of computation such as Crutchfield and Young (1990) and Langton (1990) would lead one to suspect that larger entropy would lead to better signal reproduction performance.

**Figure 3:**
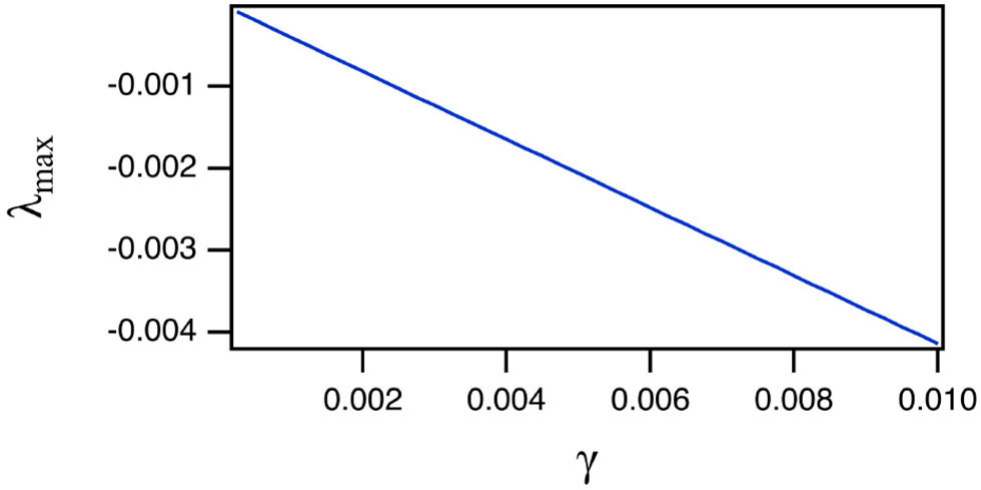
Largest Lyapunov exponent λ_*max*_ for the spiking reservoir when driven by the Sprott A system as the damping constant γ varies. The largest Lyapunov exponents when driven by the other systems were almost identical, so only the result from A is shown.

The reason for the large training error Δ_*CC*_ is explained by Figure 3, which shows the largest Lyapunov exponent for the spiking reservoir computer as γ varies. The Lyapunov exponent was calculated by the Gram-Schmidt method (Parker and Chua, 1989). The value of this Lyapunov exponent ranges from −1 × 10^−4^ to −4 × 10^−3^. This range may be compared with the Lyapunov exponents for the Sprott systems in Table 1. For all four Sprott systems the largest Lyapunov exponent for the spiking reservoir computer is larger than the negative Lyapunov exponent for each of the Sprott systems. It has been shown in Badii et al. (1988) that such an overlap can increase the fractal dimension of a signal, and Carroll (2020a) showed that this dimension increase can lead to an increase in training error.

### 6.2. Varying *R*_τ_

**Figure 4:**
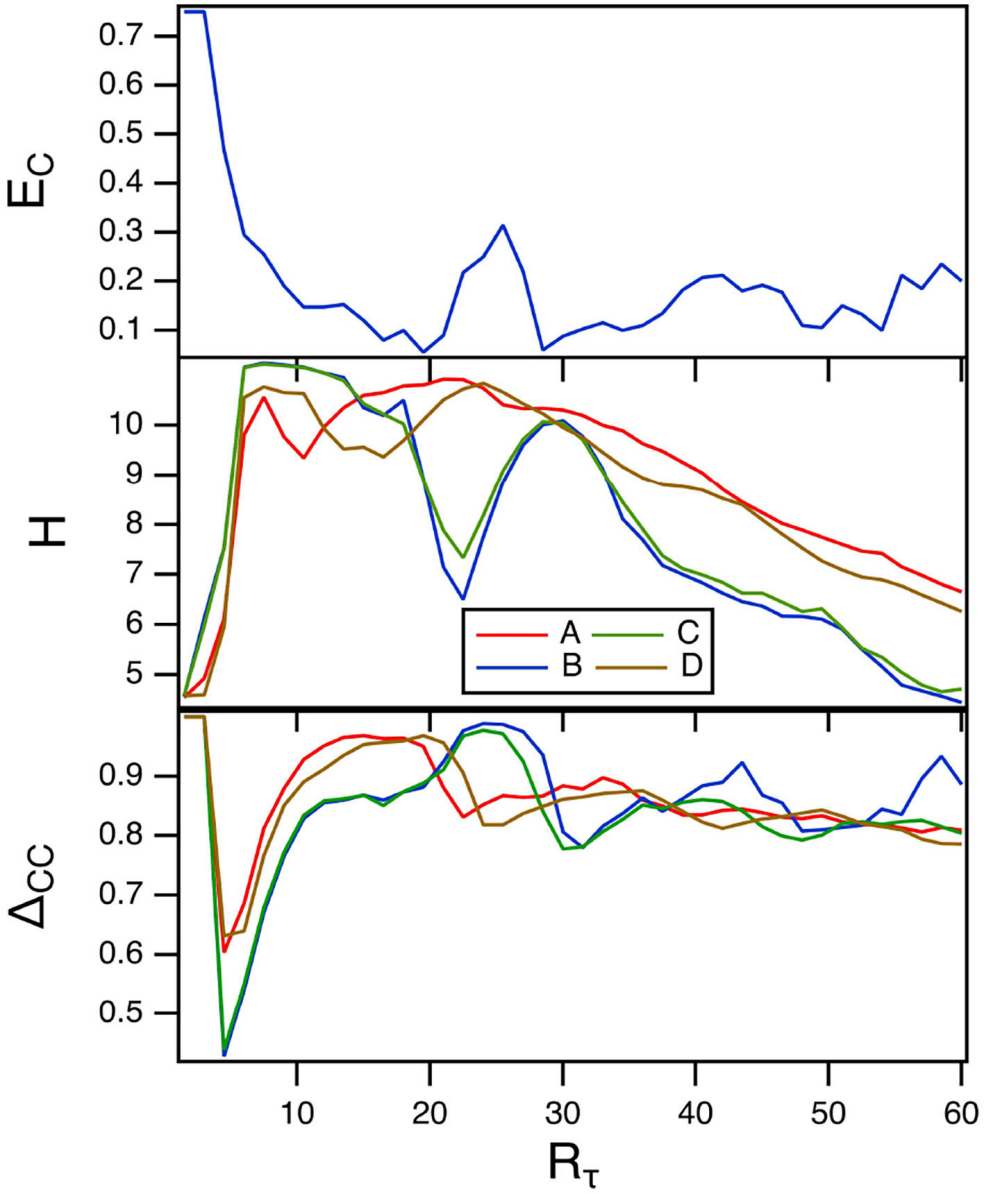
The top plot shows the fraction of errors *E*_*C*_ in identifying which of the four Sprott systems was present, as a function of the fast to slow time ratio *R*_τ_ for the spiking reservoir computer. The middle plot shows the entropies for the four Sprott systems over the same parameter range. The bottom plot is the training error Δ_*CC*_. For this plot, γ = 0.004 and *R*_τ_ = 36.

The parameter *R*_τ_ in the spiking nodes sets the ratio between fast and slow times. The top plot in Figure 4 is the fraction of errors in classifying the Sprott signals as *R*_τ_ is varied. The other parameters for this plot were γ = 0.004 and *T*_*C*_ = 15. The middle plot of this figure shows the reservoir computer entropy. The lowest error fraction comes when the reservoir computer entropy is larger, as was also seen when γ was varied. The training error Δ_*CC*_ is in the bottom plot. The training error is large over the entire range of *R*_τ_, so it appears that values of *R*_τ_ that minimize the classification error fraction do not result in small training errors. As in the previous section the overlap between the reservoir computer Lyapunov spectrum and the Lyapunov exponents of the Sprott systems may be responsible for this large error. The largest Lyapunov exponent for the spiking reservoir computer for this range of parameters ranges from –0.04 to 0.

#### 6.2.1. Effect of Window Length on Entropy

**Figure 5:**
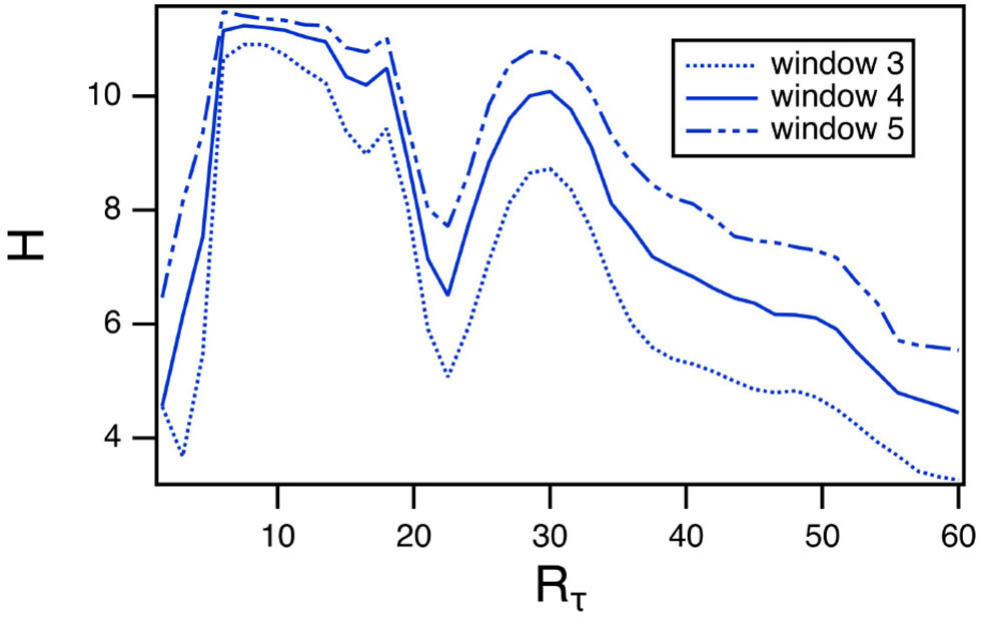
Permutation entropy *H* calculated using different window lengths as a function of the fast to slow time ratio *R*_τ_ for the spiking reservoir computer driven by Sprott system B. For this plot, γ = 0.004 and *R*_τ_ = 36.

The length of the window in the permutation entropy calculation is an arbitrary parameter, so it is reasonable to ask how changing this parameter affects the results. The curve of entropy vs. *R*_*tau*_ for Sprott system B has a very distinct pattern, so the permutation entropy calculation was repeated with window lengths of 3 and 5. The results are shown in Figure 5. The entropy does get larger as the window length increases, but the pattern remains the same as *R*_τ_ varies.

### 6.3. Varying *T*_*C*_

**Figure 6:**
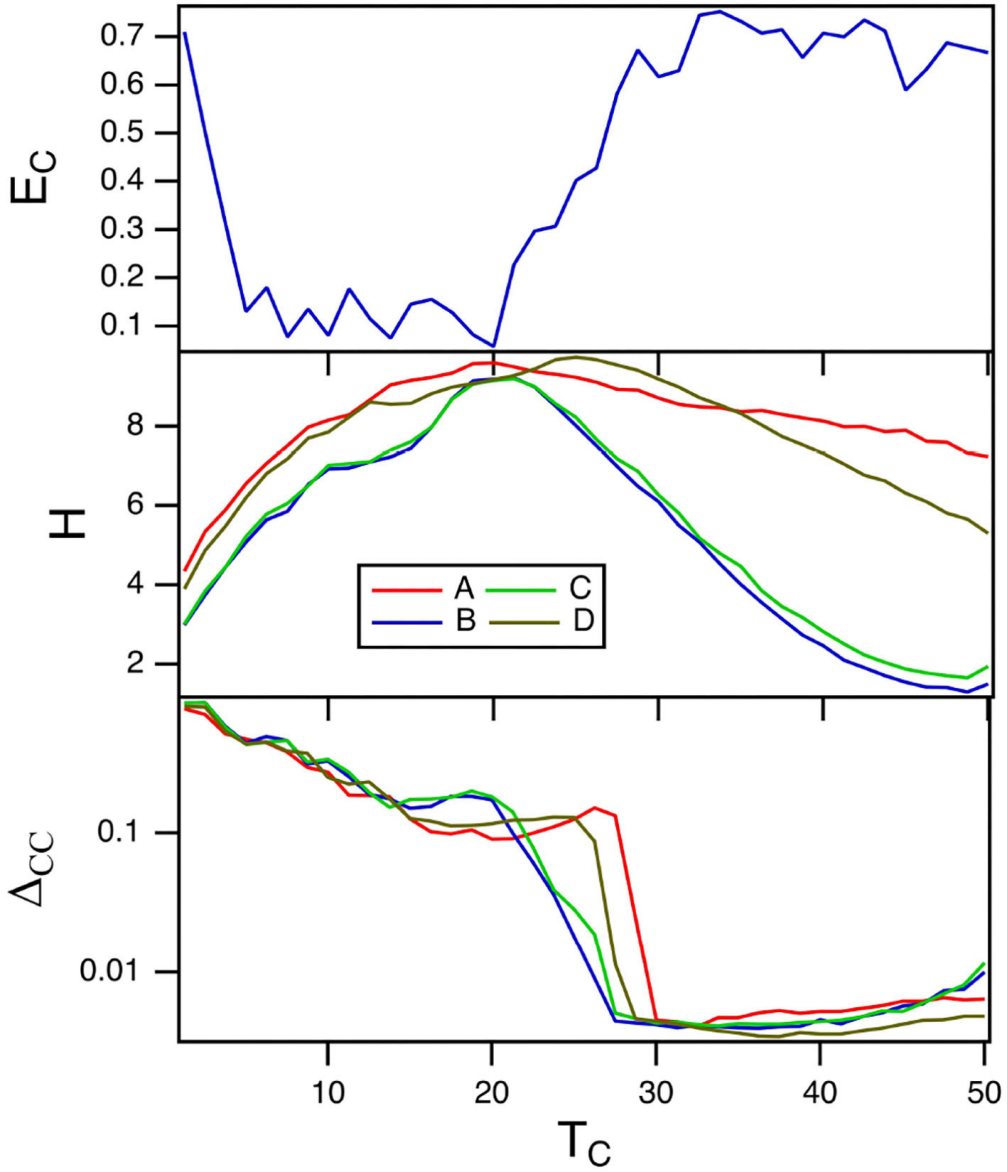
The top plot shows the fraction of errors *E*_*C*_ in identifying which of the four Sprott systems was present, as a function of the time constant *T*_*C*_ for the spiking reservoir computer. The middle plot shows the entropies *H* for the four Sprott systems over the same parameter range. The bottom plot is the training error Δ_*CC*_. For this plot, γ = 0.004 and *R*_τ_ = 36.

The parameter *T*_*C*_ for the spiking nodes matches the time scale of the reservoir computer to the time scale of the input signal. Figure 6 shows the fraction of errors for the spiking reservoir when identifying the Sprott signals (top plot) and the reservoir computer entropy (middle plot). The training error is shown in the bottom plot. The error fraction *E*_*C*_ is small for a range of values of *T*_*C*_, but it is useful to note that the region of largest entropy *H* corresponds to a low error fraction.

The plot of the training error in almost looks like the inverse of the plot of error fraction. The training error Δ_*CC*_ is large where *E*_*C*_ is small and smaller where *E*_*C*_ is larger. The conventional picture of computing with dynamical systems (Crutchfield and Young, 1990; Langton, 1990) suggests that the training error should be smaller when the entropy is large, but Figure 6 shows that in this situation the opposite is true.

As when γ was varied, the maximum Lyapunov exponent for the spiking reservoir computer affects the training error. The reservoir maximum Lyapunov exponent varies between −6 × 10^−3^ and zero as *T*_*C*_ varies, so the reservoir Lyapunov spectrum overlaps with the Sprott Lyapunov spectra, causing a larger training error.

These simulations of the spiking reservoir computer have shown that the best performance in classifying signals comes when the entropy of the reservoir computer is large. This is not surprising, as good signal identification depends on maximizing the difference in the response of the reservoir to different signals. What is surprising is that the performance in fitting the input signal is poor when the classification performance is good. It was shown in Carroll (2020a) that the highest entropy in a reservoir computer sometimes occurs when the Lyapunov exponent is approaching zero. This is expected (Crutchfield and Young, 1990; Langton, 1990), but as the Lyapunov exponent approaches zero it may also overlap with the Lyapunov spectrum of the input system, increasing the fractal dimension of the reservoir signals, resulting in poor signal fitting performance.

To optimize the signal classification performance of a reservoir computer without computing enough realizations to get good error statistics, it would be most useful to adjust the reservoir computer parameters to maximize the entropy. This process should only require one realization of the reservoir computer for each set of parameters, rather than many.

**Figure 7:**
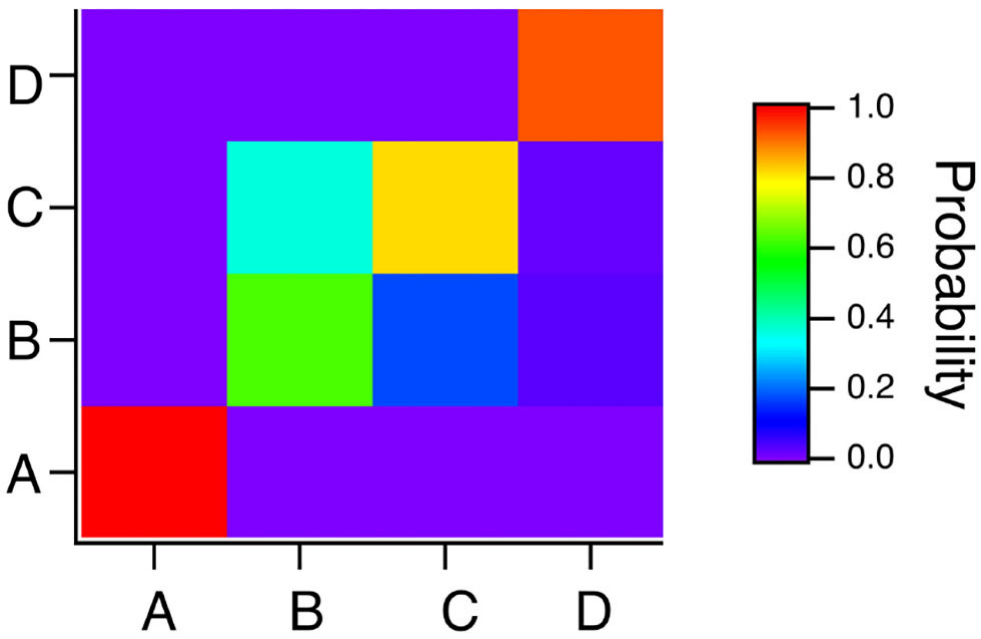
Confusion matrix for identifying the four Sprott systems A,B,C and D with the spiking reservoir computer when γ = 0.004, *R*_τ_ = 36 and *T*_*C*_ = 15. Along the *x* axis are the actual Sprott systems, while the *y* axis shows the probability that the Sprott system will be identified as that particular system.

To summarize the results for the spiking reservoir computer for the four Sprott systems, Figure 7 is a confusion matrix for this type of reservoir computer for γ = 0.004, *T*_*C*_ = 15 and *R*_τ_ = 36. The confusion matrix shows that system A is always identified correctly, but systems B and C can be hard to distinguish. System D is usually identified correctly, but it is sometimes identified as system B.

The spiking reservoir computer in this section uses a simple set of equations with a finite range of parameters. The significance of this model to biology, or to reservoir computers in general, is not known. To determine if the results in this section apply in general, a very different reservoir computer is simulated in the next section.

## 7. Continuous Nodes

The spiking nodes in the previous sections may not be a completely accurate model of a biological system, and the results may only be true for those particular nodes. To see if the results in the previous sections are general, a different type of node is used to create a reservoir computer. If similar results are seen for these very different nodes, then the results are more likely to apply to reservoir computers in general.

The nodes in the continuous reservoir computer are a polynomial ordinary differential equations (ODE) described by

(12) $$\begin{array}{l} \frac{dr_{i}\left(t\right)}{dt}\;=\alpha \left[p_{1}r_{i}\left(t\right)+p_{2}r_{i}^{2}\left(t\right)+p_{3}r_{i}^{3}\left(t\right)+\overset{M}{\underset{j=1}{\sum }}A_{ij}r_{j}\left(t\right)+W_{i}s\left(t\right)\right]. \end{array}$$

There were *M* = 100 nodes in total. This type of node was introduced in Carroll and Pecora (2019).

As in the previous sections, the adjacency matrix **A** was created by randomly selecting half of its entries and setting them to values drawn from a uniform random distribution between –1 and 1 and the diagonal elements of **A** were then set to zero. Unlike the previous section, no offset was added to the adjacency matrix. The adjacency matrix was renormalized to have a specified spectral radius σ. The elements of input vector **W** were again chosen from a uniform random distribution between –1 and 1. The polynomial ODE was numerically integrated with a fourth order Runge-Kutta routine with a step size of 1.

In the training stage, the input signal *s*(*t*) was the *x* signal from one of the four Sprott systems (A,B,C,D). The Sprott systems in this section were numerically integrated with a time step of 0.1. The first 1,000 points from the *r*_*i*_(*t*) time series are discarded and the next 20,000 points are used to fit the training signal. The actual fit signal is $h\left(t\right)=\sum_{i=1}^{M}c_{i}r_{i}\left(t\right)$, where the fit is usually done by a ridge regression to avoid overfitting. The training error is

(13) $$\begin{array}{l} \Delta_{RC}=\frac{\langle x\left(t\right)-h\left(t\right)\rangle }{\langle x\left(t\right)\rangle } \end{array}$$

where 〈〉 indicates a standard deviation.

The result of the training was a set of fit coefficients **C**_α_, α = A,B,C, or D.

For testing the classification procedure, 100 instances of the *x* signal of length 2,000 points were used to drive the polynomial ODE reservoir computer. The sets of fit coefficients for the different Sprott systems in the testing phase were designated **K**_β_, β = A,B,C, or D. As in the previous sections, the classification error was found by taking the Euclidean difference between the coefficients from the training and testing stages. For example, if β = A, the errors δ_*b*_ were found as

(14) $$\begin{array}{l} \delta_{b}=\sqrt{\overset{M}{\underset{l=1}{\sum }}{\left(K_{l,A}-C_{l,b}\right)}^{2}}b=\text{A},\text{B},\text{C},\text{D} \end{array}$$

where *l* indicates the particular component of the coefficient. If the value of *b* corresponding to the minimum δ_*b*_ is not equal to A, then an error is recorded.

### 7.1. Variation of *p*_1_

**Figure 8:**
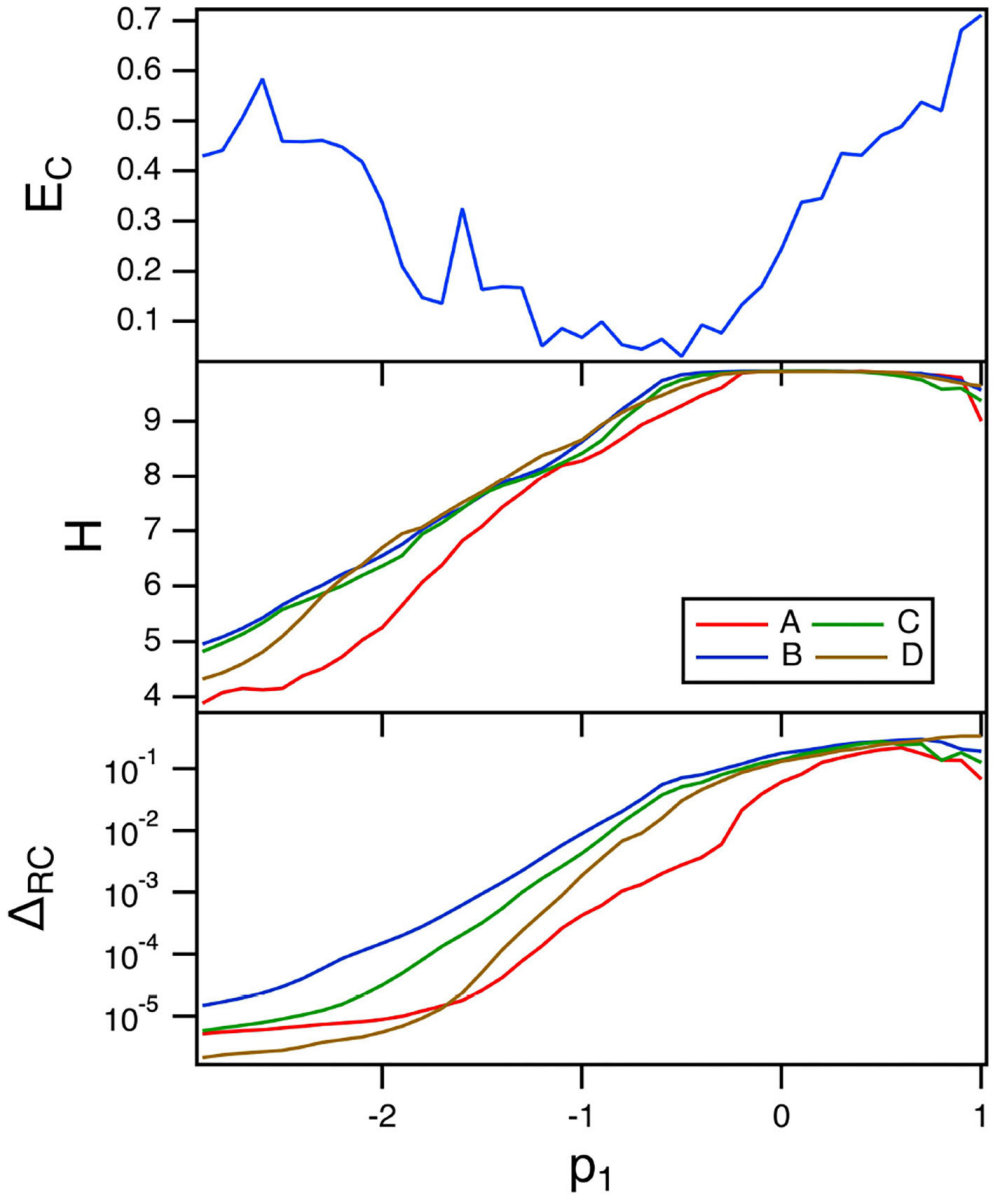
The top plot shows the fraction of errors *E*_*C*_ in identifying which of the four Sprott systems was present, as a function of the linear parameter *p*_1_ for the polynomial ODE reservoir computer. The middle plot shows the entropies for the four Sprott systems over the same parameter range. The bottom plot shows the training error Δ_*RC*_. For this plot, α = 0.3 and the spectral radius σ = 0.8.

The top plot in Figure 8 is a plot of the fraction of errors in identifying the four Sprott systems as the linear parameter *p*_1_ in the polynomial ODE reservoir is varied. For this plot, α = 0.3 and the spectral radius σ = 0.8.

The minimum error in identifying the four Sprott systems using the polynomial ODE reservoir computer comes at a value of *p*_1_ near the maximum reservoir computer entropy. This is the same behavior the was seen for the spiking reservoir computer. The best parameter for classifying the Sprott signals is not the best parameter for fitting the Sprott *x* signal, as Figure 8 shows.

The training error when *p*_1_ varies is large even though the entropy of the reservoir computer is near its maximum. Once again, the maximum Lyapunov exponent for the reservoir computer is large enough to overlap with the Lyapunov spectrum of the Sprott systems, causing an increase in the fractal dimension of the reservoir signals. The largest Lyapunov exponent for the polynomial reservoir computer as *p*_1_ is varies ranges between –0.7 and 0. Table 1 shows that the reservoir computer Lyapunov exponent overlaps with the Lyapunov spectra of systems B,C and D, and overlaps with the Lyapunov spectrum of system A for part of the range of *p*_1_.

### 7.2. Variation of α

**Figure 9:**
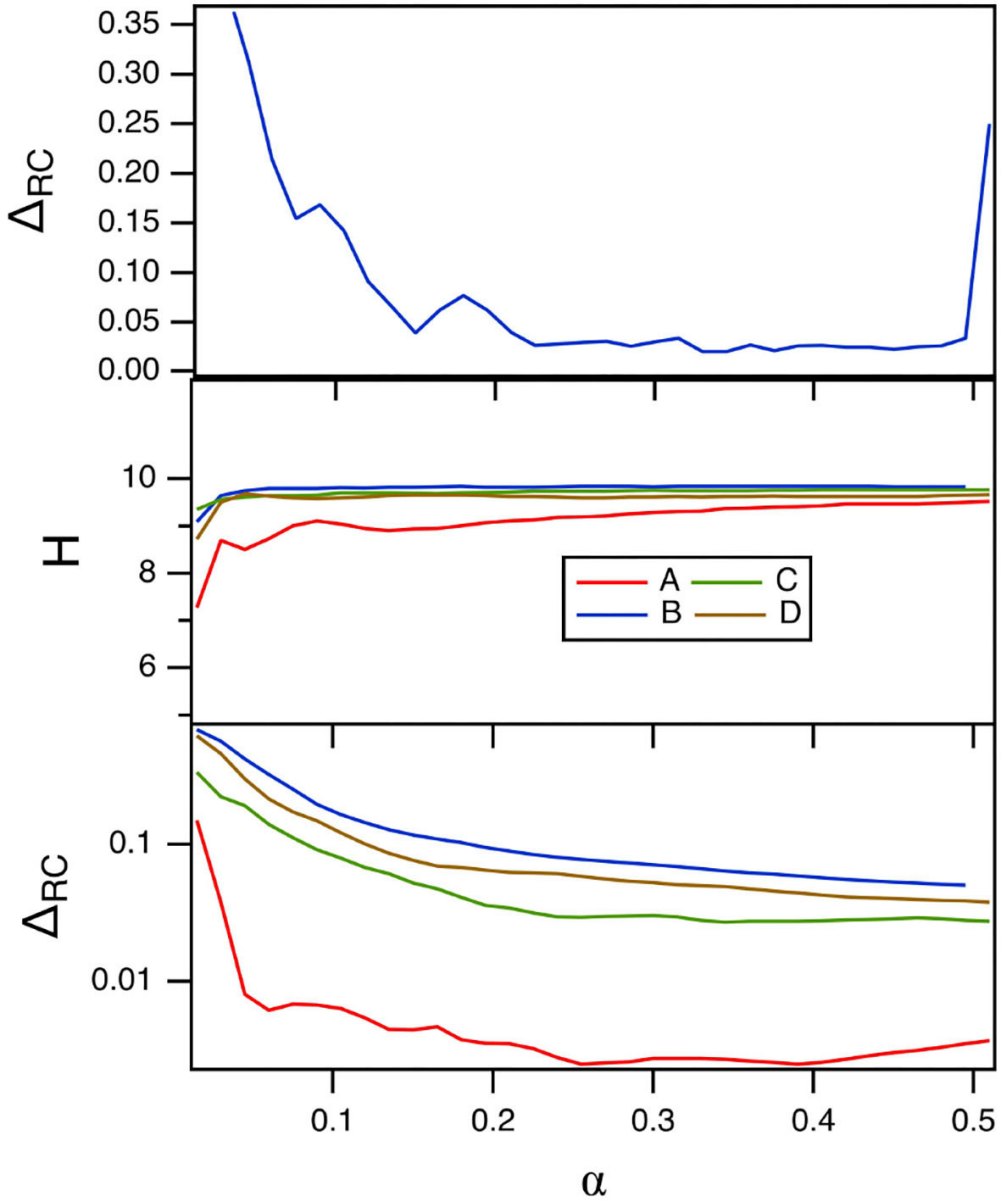
The top plot shows the fraction of errors *E*_*C*_ in identifying which of the four Sprott systems was present, as a function of the time scale parameter α for the polynomial ODE reservoir computer. The middle plot shows the entropies for the four Sprott systems over the same parameter range, while the bottom plot is the training error Δ_*RC*_. For this plot, *p*_1_ = −0.5 and the spectral radius σ = 0.8.

The parameter α varies the time scale of the polynomial ODE reservoir computer. The main effect of α is to match the frequency response of the reservoir computer to the frequency spectrum of the input signal. Figure 9 shows the fraction of errors in identifying the four Sprott systems, *E*_*C*_ in the top plot, and the reservoir computer entropy *H* in the middle plot. The bottom plot is the training error. For this figure, *p*_1_ = −0.5 and σ = 0.8.

Figure 9 shows that the error in identifying the Sprott systems is low for most of the range of α. For α≥0.5, the polynomial ODE reservoir computer becomes unstable. The reservoir computer entropy is high over the entire range of α. The training error as α is varied appears to be small, but comparing with the training error shown in Figure 8 shows that the training error is not as small as it could be for the polynomial ODE reservoir computer. Once again, the Lyapunov exponent spectrum of the reservoir computer overlaps the Lyapunov exponent spectrum of the Sprott systems. The largest Lyapunov exponent for the reservoir computer for these parameters ranges from –0.05 to 0, numbers that may be compared to the Sprott Lyapunov exponents in Table 1.

### 7.3. Variation of Spectral Radius σ

**Figure 10:**
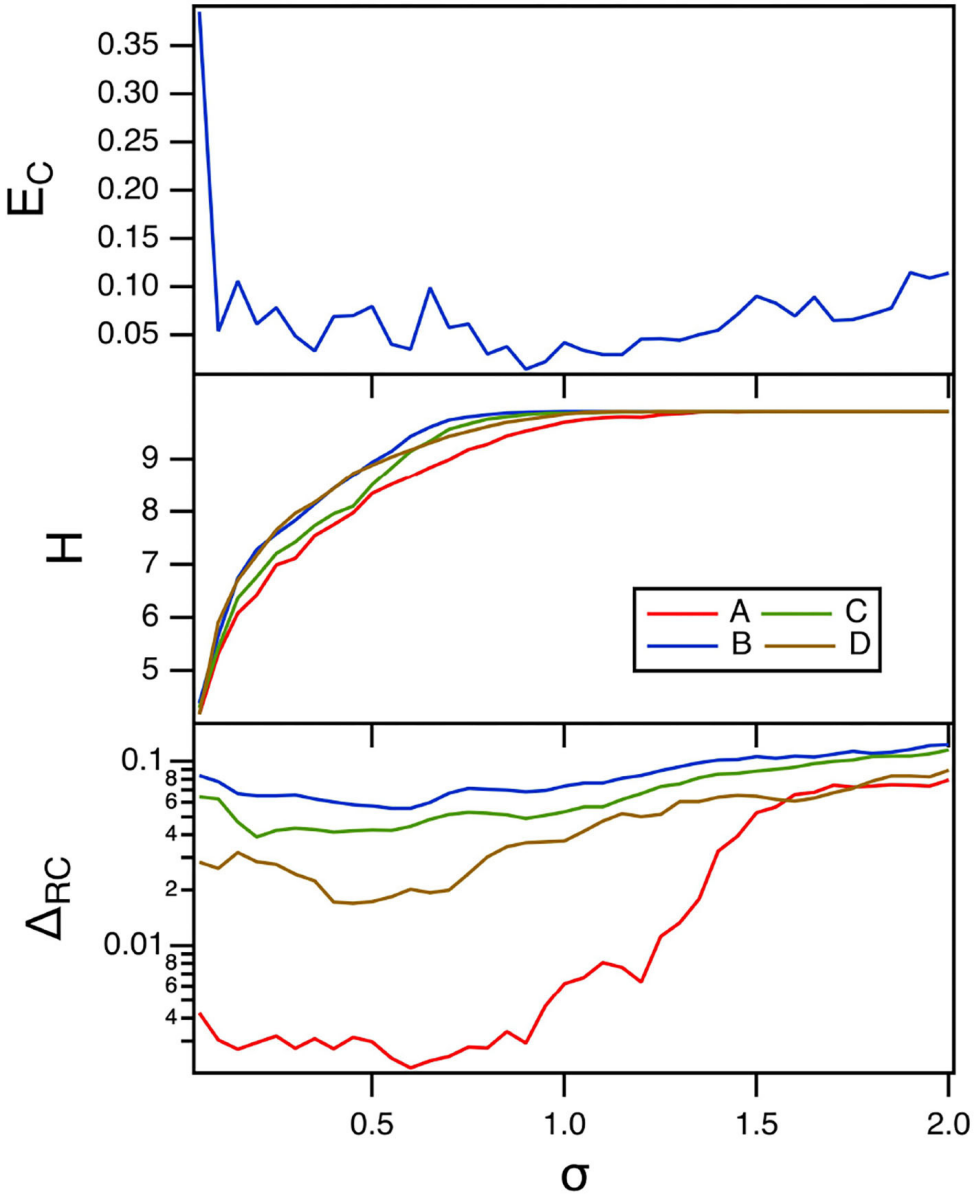
The top plot shows the fraction of errors *E*_*C*_ in identifying which of the four Sprott systems was present, as a function of the spectral radius σ for the polynomial ODE reservoir computer. The bottom plot shows the entropies for the four Sprott systems over the same parameter range. The training error Δ_*RC*_ is shown in the bottom plot. For this plot, *p*_1_ = −0.5 and the time constant *T*_0_ = 0.3.

The final parameter to be varied for the polynomial ODE reservoir computer is the spectral radius σ of the adjacency matrix. Carroll (2020b, 2021b) showed that increasing the interaction between reservoir computer nodes by increasing the spectral radius increased the entropy. The top plot in Figure 10 shows the error fraction in identifying the Sprott systems as the spectral radius is swept. The time constant α for this plot was 0.3, while the parameter *p*_1_ was –0.5. The middle plot in this figure shows the entropy of the reservoir computer.

There is a broad range of σ in Figure 10 for which the identification error is small even though the entropy varies considerably. Nevertheless, having a large value of entropy still leads to small identification error, so maximizing the entropy is a useful way to minimize the identification error.

The training error for the polynomial reservoir computer is plotted in the bottom plot of Figure 10. While the training error is not large, it does not get as small as for the lowest values of *p*_1_ in Figure 8. The largest Lyapunov exponent for the reservoir computer for this range of σ ranges from –0.12 to –0.01. Comparing to Table 1, the largest Lyapunov exponent for the reservoir computer overlaps with the Lyapunov spectrum for Sprott systems B, C, and D, but not for system A, which may be why the training error for system A is lower than for the other three systems.

#### 7.3.1. Vary Window Length

**Figure 11:**
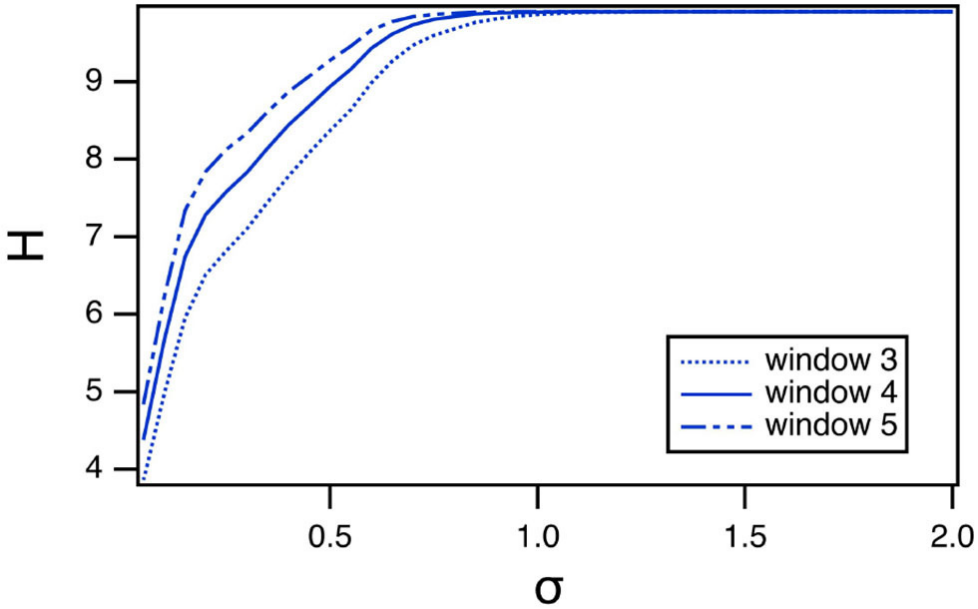
Permutation entropy *H* as a function of the spectral radius σ for the polynomial ODE reservoir computer driven by Sprott system B, as the window length varies. For this plot, *p*_1_ = −0.5 and the time constant *T*_0_ = 0.3.

As with the spiking reservoir computer, it is possible that the window length used to calculate the permutation entropy can affect the results. Figure 11 shows how the window length affects the value of the permutation entropy for Sprott system B, as the spectral radius varies. The magnitude of the permutation entropy increases with window length, but the pattern of variation does not change.

**Figure 12:**
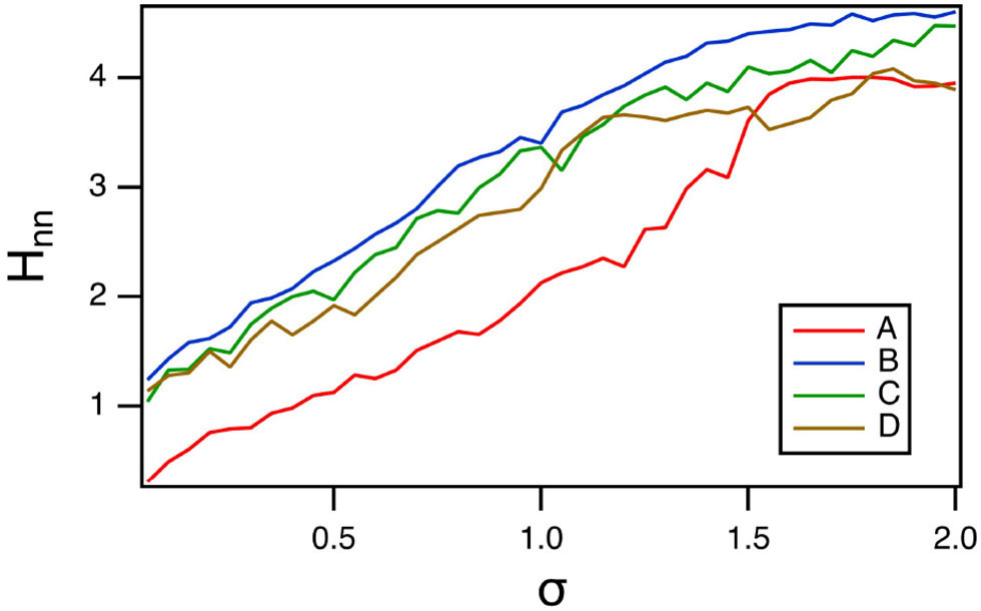
Nearest neighbor entropy *H*_*nn*_ as a function of the spectral radius σ for the polynomial ODE reservoir computer driven by Sprott systems A,B,C, and D, as the window length varies. For this plot, *p*_1_ = −0.5 and the time constant *T*_0_ = 0.3.

Because the polynomial ODE reservoir computer did not have a large a variation in time scales as the spiking reservoir computer, entropy methods that used coarse graining in phase space could also be used. In Figure 12, the nearest neighbor entropy (Kraskov et al., 2004) is estimated. For this entropy, the set of reservoir signals *r*_*i*_(*t*), *i* = 1…*M* is considered as an *M* dimensional vector **R**(*t*). A number of index points on the vector are randomly chosen, and for each index point **R**(*t*_*n*_), the distance to the *k* nearest neighbor is ε_*n*_. The nearest neighbor entropy *H*_*nn*_ is then calculated as

(15) $$\begin{array}{l} H_{nn}=\psi \left(N\right)-\psi \left(k\right)+\langle \ln\;\varepsilon_{n}\rangle \end{array}$$

where ψ is the digamma function, *N* is the number of points in the multidimensional time series and < > indicates a mean. The distances ε_*n*_ are normalized by the standard deviation of **R**(*r*), and the number of neighbors was *k* = 10.

Comparing with Figure 10, larger values of the nearest neighbor entropy correspond to smaller values of the classification error *E*_*C*_. Figure 12 shows that different methods of calculating entropy are useful for minimizing the classification error.

## 8. Conclusions

**Figure 13:**
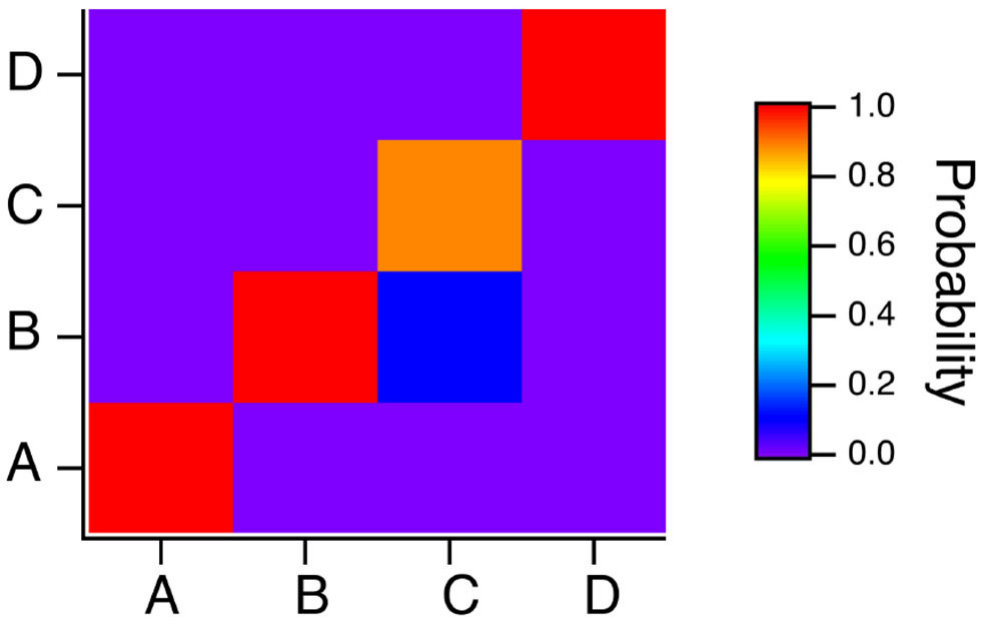
Confusion matrix for identifying the four Sprott systems A,B,C and D with the polynomial ODE reservoir computer when *p*_1_ = −0.5, the time constant *T*_0_ = 0.3 and the spectral radius σ = 0.8. Along the *x* axis are the actual Sprott systems, while the *y* axis shows the probability that the Sprott system will be identified as that particular system.

The classification results for the polynomial ODE reservoir computer are summarized in the confusion matrix of Figure 13 for which *p*_1_ = −0.5, the time constant α = 0.3 and the spectral radius σ = 0.8. Systems A and B are identified without error, but the reservoir computer has trouble distinguishing systems B and C. Figure 7 showed that the spiking reservoir computer also had trouble with these same two systems.

While it may not be practical to use a full nonlinear optimization to optimize the reservoir computer parameters for classifying time series signals, sweeping the parameters one at a time yields useful insights. It was noted for both a spiking reservoir computer and a reservoir computer based on a polynomial ordinary differential equation that the best classification performance coincides with the largest entropy for the reservoir computer. This result is not unexpected, but it is useful to confirm it using a simple method to determine entropy. The best classification performance did not always occur at the best parameters for signal reproduction.

The entropy concept used here appears to be useful, but Kanders et al. (2017) studied different measures of complexity in a spiking neural network and found that the most complex (or critical) states did not always occur when the network Lyapunov exponent was largest, so the principles found here for getting the best performance from a reservoir computer may not hold in all cases.

These results open up the possibility of optimizing a reservoir computer for signal classification by maximizing the entropy of the reservoir computer based on a single realization of the signal to be classified. Optimizing based on the classification error would require many, possibly hundreds of realizations of the signals to be classified, requiring considerable computation.

It was shown in Carroll (2020a,b) that increasing the interaction between reservoir computer nodes can increase the entropy. If the interaction is too strong, the reservoir computer may become chaotic or unstable, in which case the reservoir computer signals may increase without bound. Carroll (2021b) showed that instability may be avoided by adjusting other reservoir computer parameters so that the largest Lyapunov of the reservoir computer remains negative.

Other methods of parameter optimization in reservoir computers, such as Yperman and Becker (2017), will also work as long as the optimization criteria used is the entropy.

Neither of the reservoir computers in this study were designed with biological relevance in mind, but the fact that the requirements for the best classification performance in both types of reservoir computer were similar means that biological systems may also work best for classifying inputs when their entropy is maximized.

## Data Availability Statement

The original contributions presented in the study are included in the article/supplementary material, further inquiries can be directed to the corresponding author/s.

## Author Contributions

The author confirms being the sole contributor of this work and has approved it for publication.

## Conflict of Interest

The author declares that the research was conducted in the absence of any commercial or financial relationships that could be construed as a potential conflict of interest.
